# Improved glycemic control with proximal intestinal bypass and weight loss following gastrectomy in non-obese diabetic gastric cancer patients

**DOI:** 10.18632/oncotarget.22262

**Published:** 2017-11-01

**Authors:** Ali Guner, Minah Cho, Taeil Son, Hyoung-Il Kim, Sung Hoon Noh, Woo Jin Hyung

**Affiliations:** ^1^ Department of Surgery, Yonsei University College of Medicine, Seoul, Republic of Korea; ^2^ Department of General Surgery, Karadeniz Technical University College of Medicine, Farabi Hospital, Trabzon, Turkey; ^3^ Gastric Cancer Center, Yonsei Cancer Center, Yonsei University Health System, Seoul, Republic of Korea

**Keywords:** gastric cancer, gastrectomy, diabetes, proximal intestinal bypass, weight loss

## Abstract

**Purpose:**

The aim of this study was to assess whether gastrectomy influences glycemic control in non-obese diabetic gastric cancer patients and to identify factors related to glucose metabolism after gastrectomy.

**Materials and Methods:**

We retrospectively analyzed changes in glucose metabolism in 238 non-obese (body mass index < 30 kg/m^2^) patients with type II diabetes who underwent distal gastrectomy with either gastroduodenostomy (*n =* 91) or gastrojejunostomy (*n =* 147) for stage I gastric cancer. We collected demographics, diabetes-related features, surgery-related features, and changes in glucose metabolism during follow-up. The effect of surgery on the course of diabetes was evaluated at different time points according to fasting blood glucose levels and use of diabetes-related medication.

**Results:**

Preoperatively, the mean body mass index was 24.3 ± 2.3. Weight, body mass index and fasting blood glucose of all patients were significantly lower compared to preoperative levels at all time points. Weight loss after 6 months and the percentage of patients whose weight loss ratio was higher than 10% after one year were greater in the gastrojejunostomy group than the gastroduodenostomy group. Overall, 88 (37%) patients showed improvement in their diabetes course at one month after surgery; 152 (64%) showed improvement after 2 years. Duration of diabetes, weight loss, and reconstruction type were associated with improvement in diabetes at different time points. At 6 months and thereafter, the percentage of patients with an improved diabetes course was highest in the gastrojejunostomy plus higher than 10% weight loss group.

**Conclusions:**

Although weight loss may be associated with adverse effects of gastrectomy, postoperative weight loss in an acceptable range is a useful measure of the better glycemic control for the group of diabetic patients. Selecting gastrojejunostomy during gastrectomy and inducing acceptable weight loss after gastrectomy could be beneficial to the non-obese diabetic gastric cancer patients for improved glycemic control.

## INTRODUCTION

Development of diagnostic tools and use of mass screening program in East Asia contributed increased early detection and resulted in prolonged survival of gastric cancer patients [1–3]. Comorbid disease has emerged as the leading cause of mortality in gastric cancer patients, rather than cancer itself, especially at earlier stages [4–5]. Impairing physico-emotional health and contributing to lifestyle limitations, type II diabetes mellitus (DM) is the most common comorbidity in gastric cancer patients, with an increasing incidence that has already surpassed 15% [6]. Although diet, exercise, and pharmacotherapy are the primary therapeutic options for treating this progressive disease, surgery has recently been recognized as an effective treatment modality for managing DM [7].

Bariatric surgery was developed as a treatment for helping obese individuals achieve weight loss. Subsequent studies discovered further benefits for bariatric surgery in patients with DM, including improved insulin sensitivity, normalization of plasma glucose levels, and rapid declines in insulin requirements [8–9]. These observations ushered in the use of surgical interventions to manage DM in non-obese individuals, deemed metabolic surgery. Follow-up studies of metabolic surgery in non-obese individuals highlighted improvements in glycemic control similar to those in obese patients after bariatric surgery [10–11].

Metabolic surgery and gastric cancer surgery both result in reduced gastric volume and anatomical reconstruction of the stomach. Additionally, studies suggest that, like metabolic surgery, gastrectomy also elicits favorable changes in glucose metabolism in diabetic gastric cancer patients after surgery [12–14]. However, these studies included patients with advanced stage cancer who received postoperative chemotherapy or chemoradiotherapy or patients who underwent total gastrectomy, which could potentially affect glycemic control. Also, obese patients were included to the studies. Thus, the pure impact of gastrectomy on glucose metabolism in non-obese diabetic gastric cancer patients has not been accurately evaluated.

Herein, we hypothesized that physiological changes in food passage after gastrectomy, together with changes in weight, could influence glucose metabolism in non-obese diabetic gastric cancer patients. Accordingly, we designed this study to assess the effects of changes in food passage and weight on glucose metabolism according to glycemic profiles and to identify an appropriate procedure for surgical reconstruction of the stomach after gastrectomy in gastric cancer patients with type II DM.

## RESULTS

### Patient characteristics

Of the 238 patients, 179 (75%) were male and 59 (25%) were female. The mean age was 62.9 ± 8 years. The median duration of DM was 8 years (range, 0–36). Only 42 patients (17%) had not used any antidiabetic medication, while the other 196 patients (83%) were prescribed an oral antidiabetic agent, insulin, or both. Preoperatively, the median fasting blood glucose level was 137 mg/dl (range, 61–285 mg/dl) and the median HbA1c was 7.2 (range, 4.5–14.8). Preoperative mean weight and BMI values were 65.9 ± 8 kg and 24.3 ± 2.3, respectively. The median follow-up duration was 58 months (range, 24–97 months).

In total, 147 (62%) patients underwent Billroth-I gastroduodenostomy; 91 (38%) underwent Billroth-II gastrojejunostomy. There were no significant differences between the gastroduodenostomy and groups in terms of age, gender, BMI, BMI class, diabetes-related features, and histopathological features. Mean preoperative weights, however, were significantly different between the reconstruction groups (64.9 ± 8.3 vs 67.5 ± 7.3). The characteristics of all patients and a comparison of the patients grouped according to type of reconstruction are shown in Table 1.

**Table 1:** Characteristics of all patients and comparison of groups divided according type of gastric reconstruction

	All patients (n:238)		Billroth-I gastroduodenostomy (n: 147)	Billroth-II gastrojejunostomy (n: 91)	*p* value
Age (year)	62.9 ± 8		62.9 ± 8.1	63.03 ± 7.9	0.920
Sex					0.086
Male	179 (75%)		105 (71%)	74 (81%)	
Female	59 (25%)		42 (29%)	17 (19%)	
Duration of diabetes (years)	8 (0–36)		9 (0–36)	7.5 (0–24)	0.075
Weight (kg)	65.9 ± 8		64.9 ± 8.3	67.5 ± 7.3	0.016
Body mass index (kg/m^2^)	24.3 ± 2.3		24.1 ± 2.3	24.6 ± 2.2	0.085
Body mass index class^a^					0.210
Normal weight	142 (59%)		93 (63%)	49 (54%)	
Overweight	96 (41%)		54 (37%)	42 (46%)	
Fasting blood glucose (mg/dL)	137 (61–285)		139 (61–261)	131 (67–285)	0.415
HbA1c (%)	7.2 (4.5–14.8)		7.1 (4.5–14.8)	7.4 (5.7–10.7)	0.607
Antidiabetic treatment					0.805
No treatment	42 (17%)		27 (18%)	15 (17%)	
Oral medication	171 (73%)		107 (73%)	64 (70%)	
Insulin	12 (5%)		7 (5%)	5 (5%)	
Both	13 (5%)		6 (4%)	7 (8%)	
Histology^b^					0.059
Differentiated	159 (67%)		105 (71%)	54 (59%)	
Undifferentiated	79 (33%)		42 (29%)	37 (41%)	
T classification^c^					0.687
T1	212 (89%)		130 (88%)	82 (90%)	
T2	26 (11%)		17 (12%)	9 (10%)	
N classification^c^					0.488
N0	223 (94%)		139 (95%)	84 (92%)	
N1	15 (6%)		8 (5%)	7 (8%)	
Pathological stage^c^					0.928
Stage Ia	197 (83%)		122 (83%)	75 (82%)	
Stage Ib	41 (17%)		25 (17%)	16 (18%)	

^a^based on World Health Organization classification

^b^differentiated type includes papillary adenocarcinoma and well or moderately differentiated tubular adenocarcinoma, and undifferentiated type includes poorly differentiated adenocarcinoma, signet-ring cell carcinoma, undifferentiated carcinoma, or mucinous adenocarcinoma.

^c^based on American Joint Committee on Cancer guidelines-7th edition.

### Changes in weight, BMI, fasting blood glucose, and HbA1c

**Figure 1:**
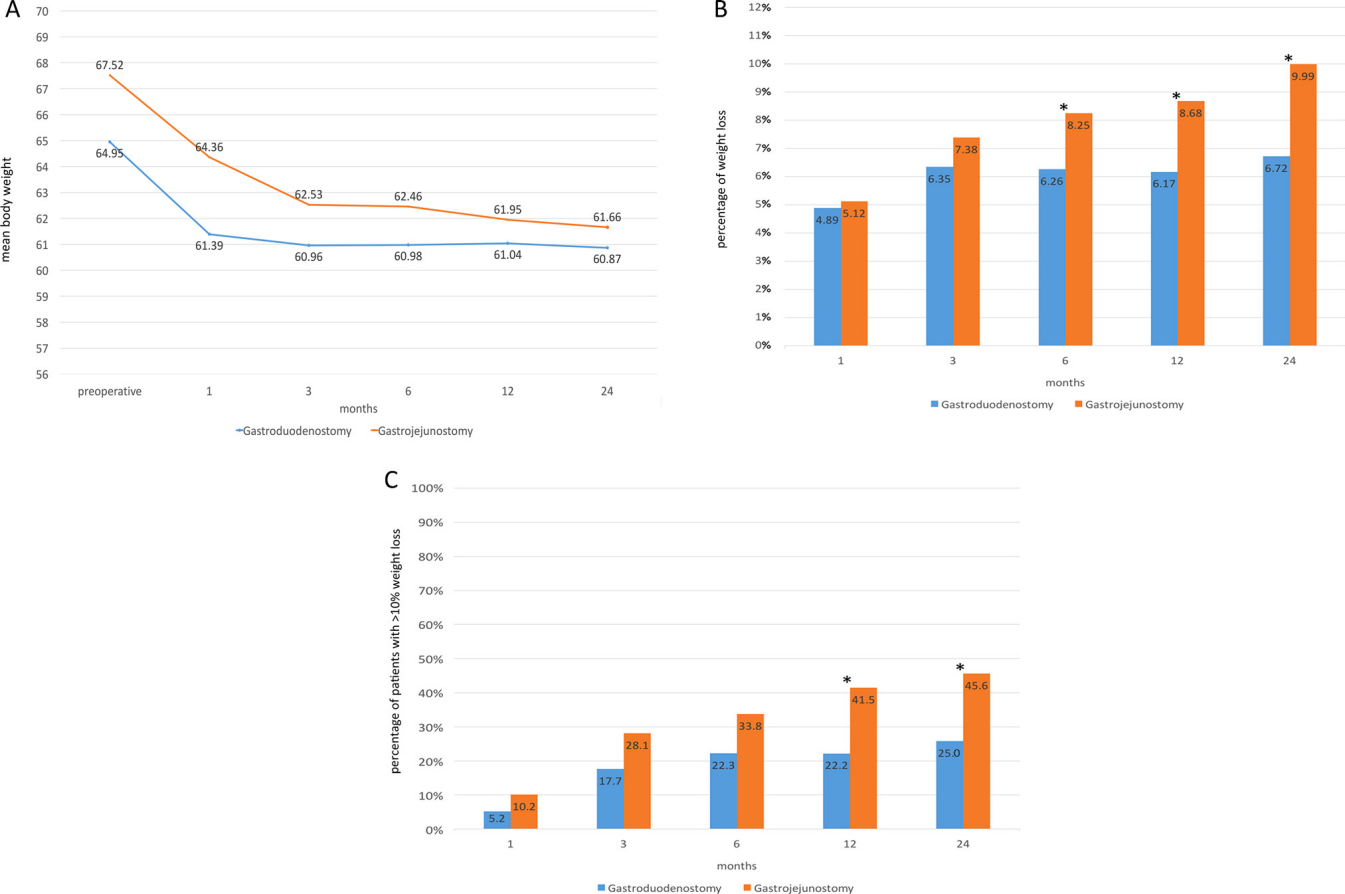
Changes in body weight (**A**), weight loss ratio compared to preoperative levels (**B**) and percentage of patients with a weight loss ratio greater than 10% during the follow-up period (**C**) according to type of reconstruction. Asterisks indicate statistically significant differences for percentages between groups. BMI: Body mass index.

Changes in weight, BMI, fasting blood glucose, and HbA1c were evaluated at 1, 3, 6, 12, and 24 months after surgery. Changes in body weight were shown in Figure 1A. Weight and BMI of all patients were significantly lower compared to preoperative levels at all time points. Compared to the Billroth I gastroduodenostomy group, patients in the Billroth-II gastrojejunostomy group showed greater weight loss after 6 months; moreover, the percentage of patients in the Billroth-II gastrojejunostomy group with a weight loss ratio higher than 10% was significantly greater at 12 and 24 months (Figure 1B–1C).

**Figure 2:**
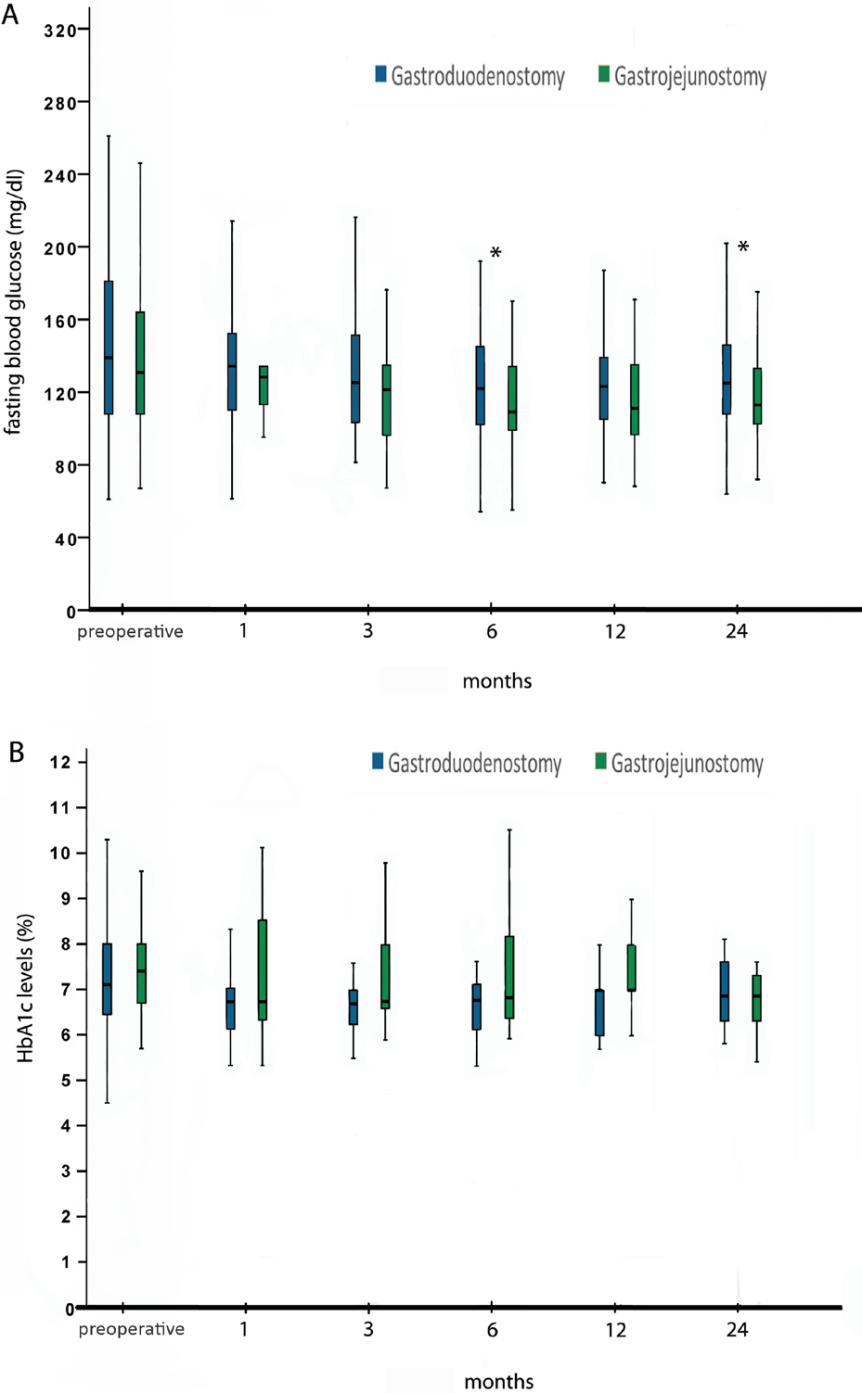
Fasting blood glucose (**A**) and HbA1c levels (**B**). Asterisks indicate statistically significant differences between groups.

Fasting blood glucose levels and HbA1c levels of both groups during the follow-up period are shown in Figure 2A–2B. In paired analysis of all patients regardless of the type of reconstruction, fasting blood glucose levels showed statistically significant decrease at all time points, and HbA1c levels at 3 (median 7.2 (4.5–14.8) vs 6.7 (5.5–10.3), *p =* 0.023) and 12 months (median 7.2 (4.5–14.8) vs 6.8 (6–9), *p =* 0.033) compared to preoperative levels. Patients in the Billroth-II gastrojejunostomy group had lower levels of fasting blood glucose at 6 and 24 months after surgery than those in the Billroth I gastroduodenostomy group. There were, however, no significant differences in HbA1c levels between the groups throughout the follow-up period.

### Factors related to improvement in DM

Improvement in glucose metabolism was evaluated at 1, 3, 6, 12, and 24 months after surgery. A total of 88 patients (37%) showed an improved course of glycemic profile at one month after surgery. The percentages of improved patients were 53% (126 patients), 60% (143 patients), 71% (169 patients), and 64% (152 patients) at 3, 6, 12, and 24 months, respectively.

Univariate analyses were conducted to identify factors contributing to changes in the course of glycemic profile. During the first and third months, no factor was found to be significantly related with improvement. By the 6th month, preoperative fasting blood glucose level, duration of DM, weight change, and type of reconstruction; by the 12th month, weight change and type of reconstruction; and by the 24th month, duration of DM, weight change, and type of reconstruction were found to be significant factors affecting improvement in glycemic profile (Table 2). In multivariate analysis, duration of DM was shown to be an independent factor for improvement at 24 months; weight change was identified as an independent factor at 6, 12, and 24 months; and type of reconstruction was found to be an independent factor at 6, 12, and 24 months (Table 3).

**Table 2:** Univariate analysis of variables affecting improvement in glycemic control at different time points after surgery

Variables	1st month		3rd month		6th month		12th month		24th month	
	OR	*p* value	OR	*p* value	OR	*p* value	OR	*p* value	OR	*p* value
Age	0.970	0.210	1.016	0.462	1.010	0.581	1.031	0.101	0.988	0.496
Sex (male* vs female)	0.667	0.815	0.902	0.791	1.165	0.640	1.099	0.780	0.795	0.468
Duration of diabetes	0.970	0.350	0.970	0.261	0.953	0.032	0.978	0.335	0.931	0.002
Preoperative weight	0.985	0.558	0.998	0.914	1.009	0.616	1.009	0.626	1.016	0.348
Preoperative BMI	0.871	0.139	0.984	0.826	1.106	0.111	1.087	0.210	1.032	0.610
Preoperative BMI class (normal* vs. overweight)	0.688	0.412	0.729	0.395	1.329	0.341	1.230	0.507	1.184	0.562
Preoperative fasting blood glucose	1.001	0.864	0.995	0.186	0.992	0.018	0.994	0.059	0.994	0.073
Preoperative HbA1c	1.091	0.699	0.671	0.057	0.759	0.062	0.822	0.163	0.826	0.166
Preoperative insulin use (no* vs. yes)	0.343	0.189	0.831	0.729	1.274	0.602	1.835	0.293	0.753	0.542
Weight change	1.164	0.997	0.939	0.171	0.909	0.004	0.920	0.008	0.907	< 0.001
Method of reconstruction (gastroduodenostomy * vs. gastrojejunostomy)	0.982	0.978	1.581	0.214	2.739	0.002	4.383	< 0.001	5.100	< 0.001

OR: Odds ratio, BMI: Body mass index, Asterisk indicates the reference variable.

**Table 3:** Multivariate analysis of variables affecting improvement in glycemic control at different time points after surgery

Variables	6th month			12th month			24th month		
	OR	95% CI	*p* value	OR	95% CI	*p* value	OR	95% CI	*p* value
Duration of diabetes	0.969	0.921–1.019	0.197	–	–	–	0.944	0.897-0.993	0.025
Preoperative fasting blood glucose	0.996	0.998–1.003	0.226	–	–	–	–	–	–
Weight change	0.922	0.860–0.989	0.024	0.931	0.873–0.992	0.028	0.929	0.877–0.983	0.011
Method of reconstruction			0.047			0.008			0.001
Gastroduodenostomy	1 (ref)			1 (ref)			1 (ref)		
Gastrojejunostomy	2.128	1.010–4.484		2.894	1.317–6.359		3.781	1.765–8.098	

OR: Odds ratio, CI: Confidence interval.

### Effects of weight changes and type of reconstruction on improvement in DM

**Figure 3:**
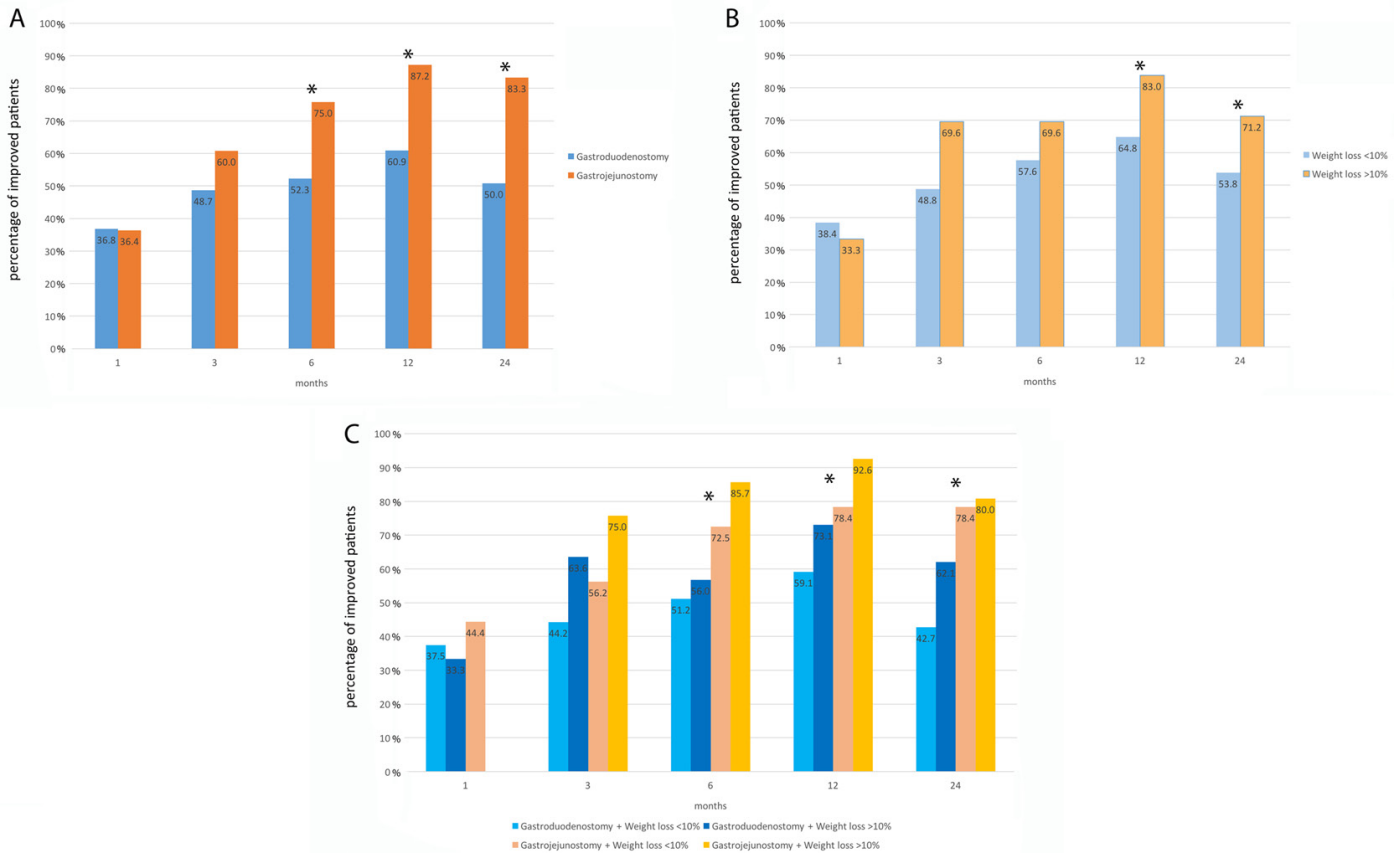
Percentages of patients with an improved course of diabetes according to reconstruction type (**A**), weight loss greater than 10% (**B**), and both reconstruction type and 10% weight loss (**C**). No patient in the gastrojejunostomy plus ≥ 10% weight loss group showed improvement during the first month. Asterisks indicate statistically significant differences between groups.

The Billroth I gastroduodenostomy and Billroth-II gastrojejunostomy groups showed no difference in their percentages of patients with improved glycemic profile for up to 6 months after surgery. Beginning in the 6th month and thereafter, the percentage of patients with improvement in glycemic control was statistically higher for Billroth-II gastrojejunostomy (Figure 3A). Meanwhile, patients experienced a weight loss ratio more than 10% showed significantly higher percentage of improvement in glycemic control at 12 and 24 months than patients experienced a weight loss ratio less than 10% (Figure 3B).

To analyze the combined effect of weight loss and type of reconstruction on glycemic profile, we categorized patients into four groups according to a weight loss ratio of less than or greater than 10%, compared to preoperative weight, and whether they underwent gastroduodenostomy or gastrojejunostomy for gastric reconstruction. The percentages of improved patients in each of the four groups are shown in Figure 3C; statistically significant differences were noted at 6, 12, and 24 months. In logistic regression analysis (reference group is gastroduodenostomy with ≤ 10% weight loss; Table 4), gastrojejunostomy plus ≥ 10% weight loss showed the best improvement (OR: 5.200, 95% CI:1.424–18.991, *p =* 0.013 at 6 months; OR: 8.654, 95% CI:1.928–38.848, *p =* 0.005 at 12 months, and OR: 5.371, 95% CI:1.984–14.541, *p =* 0.001 at 24 months). Gastrojejunostomy plus ≤ 10% weight loss showed the second-best improvement (OR: 2.285, 95% CI:1.011–5.166, *p =* 0.047 at 6 months; OR: 2.510, 95% CI:1.030–6.115, *p =* 0.043) at 12 months and OR: 4.868, 95% CI:1.986–11.934, p: 0.001 at 24 months).

**Table 4:** Logistic regression analysis of the combined effects of proximal intestinal bypass and weight loss on improved glycemic control

	6th month				12th month				24th month			
Group	*n*	OR	95%CI	*p* value	*n*	OR	95%CI	*p* value	*n*	OR	95%CI	*p* value
Gastroduodenostomy and ≤ 10% weight loss	114	1 (ref)			114	1 (ref)			110	1 (ref)		
Gastroduodenostomy and ≥ 10% weight loss	33	1.103	0.449–2.709	0.831	33	1.879	0.716–4.933	0.200	37	2.197	0.922–5.237	0.076
Gastrojejunostomy and ≤ 10% weight loss	60	2.285	1.011–5.166	0.047	53	2.510	1.030–6.115	0.043	49	4.868	1.986–11.934	0.001
Gastrojejunostomy and ≥ 10% weight loss	31	5.200	1.424–18.991	0.013	38	8.654	1.928–38.848	0.005	42	5.371	1.984–14.541	0.001

n: number of patients, OR: Odds ratio, CI: Confidence interval.

## DISCUSSION

In this study, we discovered that weight loss and type of reconstruction are associated with improvements in glucose metabolism in non-obese diabetic gastric cancer patients after gastrectomy, together with duration of DM. While weight loss resulting from decreased oral intake and reduced absorption after surgery can induce improvements in DM, duodenal bypass has been shown to improve glycemic control in type II DM patients by itself [15–17]. In the present study, nonetheless, we found that weight loss and duodenal bypass act together to improve glucose metabolism after gastrectomy in non-obese patients. Interestingly, gastrojejunostomy elicited better improvement in glycemic profile than gastroduodenostomy by facilitating greater weight loss after surgery in non-obese gastric cancer patients.

Among the non-obese patients included in this study, weight loss after surgery was found to be an independent factor for improvement in DM. For the first 6 months after surgery, weight loss amounts were comparable for gastroduodenostomy and gastrojejunostomy groups; however, in the long term, gastrojejunostomy facilitated greater weight loss, as it, unlike gastroduodenostomy, involved performing proximal intestinal bypass. Similarly, studies comparing gastric bypass or duodenal-jejunal bypass versus sleeve gastrectomy reported that bariatric procedures that bypassed the proximal intestine achieved greater weight loss than those that did not [18–20]. In accordance with the greater weight loss in the gastrojejunostomy group after 6 months, patients in this group also showed higher rates of improvement in glycemic profile. Meanwhile, however, although improvement in DM after gastric resection may be explained by weight loss, one study has suggested that multiple weight-independent mechanisms affect improvements in DM [21].

In addition to weight loss, proximal intestinal bypass, as suggested in experimental and human studies, was found to be associated with improvement in DM, along with a shorter duration of DM [22–27]. Although the exact mechanism for DM improvement after duodenal bypass is not clear, studies have demonstrated that, independent from weight loss, proximal intestinal bypass facilitates improvement in DM via incretins/anti-incretins, gut hormones or altered bile acid signaling [28–30]. Recently, endoscopically placed medical device which mimics the surgical techniques that provide duodenal bypass, has been shown effective tool for weight loss and metabolic improvement in glucose metabolism [31]. Moreover, because Billroth-II gastrojejunostomy is common in our practice as a reconstruction method after gastrectomy, we evaluated the data of Billroth-II gastrojejunostomy as the group of duodenal bypass, however, we believe Roux-en-Y reconstruction which is another reconstruction technique around the world may achieve similar outcomes.

Beyond the metabolic effects of bypassing duodenum, the length of bypassed segment of proximal intestine is an important point of metabolic surgery and the ideal extent of bypassed segment is still under debate [32]. In this study, remnant stomach was anastomosed to small bowel approximately 20 cm from the ligament of Treitz in gastrojejunostomy group patients. This length is shorter than the length of biliopancreatic limb, which is used in metabolic surgery such as Roux-en-Y gastric bypass or duodenal switch. As was shown in previous studies, we believe that the increasing of the extent of bypassed segment during gastrojejunal anastomosis in gastric cancer surgery may result with better glycemic control [33]. However, this theory should be evaluated in comparative studies.

Another finding of the present study is shorter duration of DM which is typically considered to reflect residual beta cell volume and severity of DM, has positive effect on amelioration of DM [34]. Although DM duration was not associated with improvement in glycemic profile in the early period after surgery, it was by year two. This finding suggests that diabetic patients with a shorter duration of DM and less severe disease might be more likely to experience favorable metabolic outcomes after surgery.

Our retrospective and cross-sectional analysis of the data should be considered as a limitation of this study. As definitions of improvement can vary from study to study, there are limitations to analyzing and comparing data between studies [12, 35–39]. Because we aimed to assess the glucose metabolism according to glycemic profiles in different time points, we used the term “improvement” rather than the term “remission” which requires at least one to five years follow-up period. Nevertheless, since several related conditions may alter diabetes status, we were cautious in selecting patients for inclusion in the study; we exclude individuals who were classified as obese or underweight, who received chemotherapy, who had advanced cancer, or who underwent total gastrectomy. A prospective randomized trial comparing different reconstruction procedures for gastric cancer patients with DM could potentially show which reconstruction type will allow for better improvement and may validate the data of our study. Despite the above limitations, our results provide the rationale for conducting a well-designed prospective study to identify optimal treatment strategies for diabetic patients with gastric cancer. Moreover, our analysis of non-obese patients may provide the necessary evidence with which to expand the application of metabolic surgery to non-obese diabetic patients.

In conclusion, our study showed that reconstruction type and weight loss affect glycemic control in non-obese diabetic gastric cancer patients. Patients who underwent gastrojejunostomy and were accompanied by more weight loss showed the greatest improvement in glycemic control. Although weight loss may be associated with other adverse effects of gastrectomy, we believe that postoperative weight loss in an acceptable range is a useful measure of the better glycemic control for the group of diabetic patients. Selecting gastrojejunostomy and inducing acceptable weight loss after gastrectomy could be recommendable in non-obese diabetic gastric cancer patients for improved glycemic control.

## MATERIALS AND METHODS

### Patient selection

We retrospectively reviewed data stored in a prospectively maintained gastric cancer database for patients treated at Severance Hospital of Yonsei University Health System in Seoul, Korea. We selected type II DM patients who underwent distal subtotal gastrectomy for stage I gastric cancer without a history of receiving chemotherapy. There were 252 patients who met the above criteria. Among these, two underweight patients (body mass index [BMI] < 18.5) and 12 obese patients (BMI > 30) were excluded from the analysis. Finally, we analyzed 238 non-obese, type II DM, stage I gastric cancer patients.

### Data collection

All data on patient demographics, duration of DM, reconstruction after resection, fasting blood glucose level, glycosylated hemoglobin (HbA1c), body weight, BMI, and types of antidiabetic treatment (oral antidiabetic, insulin, or both) at the time of surgery and periodically during the follow-up period were collected. Patients with fasting blood glucose levels higher than 126 mg/dl or plasma glucose levels at 2 hour after feeding higher than 200 mg/dl were diagnosed as having DM based on criteria set by the World Health Organization [40]. We divided the patients according to the World Health Organization International Classification of BMI: index values between 18.5 and 25 were defined as normal weight, values below 18.5 as underweight, and values between 25 and 30 as overweight. To analyze the effect of surgery on weight changes, we also divided patients into two groups according to weight reductions of less than or greater than 10%.

The effect of surgery on the course of glycemic profile was adapted from the American Society for Metabolic and Bariatric Surgery recommendations and was classified in one of two categories according to fasting blood glucose levels and use of diabetes-related medication [35–36]. The course of diabetes was deemed to have “improved” when patients showed a fasting blood glucose level below 126 mg/dL and either used a lower dose of antidiabetic medication or converted from insulin to oral medication. Patients who experienced no change or an aggravation in medication or fasting blood glucose levels after surgery were defined as having “not-improved”.

### Gastric reconstruction

Reconstruction after distal gastrectomy was performed with either Billroth I gastroduodenostomy or Billroth II gastrojejunostomy. While Billroth I gastroduodenostomy is relatively simple to perform, requiring only one anastomosis and allowing for maintenance of physiological intestinal continuity, it can only be performed when relatively a small part of the distal stomach is removed [41]. If achieving a safe tumor margin or acceptable anastomotic tension with gastroduodenostomy is not feasible, surgeons perform a gastrojejunostomy for reconstruction of the stomach. Billroth-II gastrojejunostomy is a gastroenterostomy technique that links the gastric pouch to the jejunum at location 15–20 cm distal to the ligament of Treitz, bypassing the proximal intestine.

### Statistical analysis

Continuous variables are presented as means ± standard deviations for a parametric distribution and medians (range) for a nonparametric distribution. The chi-square test, Mann-Whitney *U* test, and Student’s *t* test were used to compare categorical and continuous variables as appropriate. The paired analysis was fulfilled by paired *t* test or Wilcoxon signed-rank test. Univariate and multivariate analyses of variables were performed with logistic regression to identify factors associated with improvement in DM. Statistical analysis software (SPSS 20.0, IBM Corp, Armonk, NY, USA) was used to conduct all statistical analyses. All *p*-values < 0.05 were considered statistically significant.

## Abbreviations

BMI: Body mass index
DM: Diabetes mellitus
HbA1c: Glycosylated hemoglobin
